# Trauma Boot Camp: A Simulation-Based Pilot Study

**DOI:** 10.7759/cureus.463

**Published:** 2016-01-20

**Authors:** Fabiana Ortiz Figueroa, Yasmin Moftakhar, Arthur L Dobbins IV, Ramisha Khan, Rahul Dasgupta, Rachel Blanda, Tiffany Marchand, Rami Ahmed

**Affiliations:** 1 Emergency Medicine, Summa Akron City Hospital; 2 Northeast Ohio Medical University; 3 Walsh University; 4 University of Akron; 5 Georgetown University; 6 Surgical Critical Care, Summa Akron City Hospital

**Keywords:** simulation, trauma, boot camp, atls, vicarious error management, crisis resource management

## Abstract

Introduction: Interns are often unprepared to effectively communicate in the acute trauma setting. Despite the many strengths of the Advanced Trauma Life Support (ATLS) program, the main shortcoming within the course is the deficiency of teamwork and leadership training. In this study, we describe the creation of an interdisciplinary boot camp in which interns' basic trauma knowledge, level of confidence, and teamwork skills are assessed.

Methods: We designed a one-day, boot camp curriculum for interns of various specialties with the purpose of improving communication and teamwork skills for effective management of acute trauma patients. Our curriculum consisted of a one-day, twelve-hour experience, which included trauma patient simulations, content expert lectures, group discussion of video demonstrations, and skill development workstations. Baseline and acquired knowledge were assessed through the use of confidence surveys, cognitive questionnaires, and a validated evaluation tool of teamwork and leadership skills for trauma

Results: Fifteen interns entered the boot camp with an overall confidence score of 3.2 (1-5 scale) in the management of trauma cases. At the culmination of the study, there was a significant increase in the overall confidence level of interns in role delegation, leadership, Crisis Resource Management (CRM) principles, and in the performance of primary and secondary surveys. No significant changes were seen in determining and effectively using the Glasgow Coma Scale, Orthopedic splinting/reduction skills, and effective use of closed-loop communication.

Conclusion: An intensive one-day trauma boot camp demonstrated significant improvement in self-reported confidence of CRM concepts, role delegation, leadership, and performance of primary and secondary surveys. Despite the intensive curriculum, there was no significant improvement in overall teamwork and leadership performance during simulated cases. Our boot camp curriculum offers educators a unique framework to which they can apply to their own training program as a foundation for effective leadership and teamwork training for interns.

## Introduction

Upon graduation from medical school, interns are often unprepared to effectively communicate in the acute trauma setting [1-2]. Though many interns receive Advance Cardiovascular Life Support (ACLS), Pediatric Advanced Life Support (PALS), and Advanced Trauma Life Support (ATLS) training at the beginning of their residencies, there is little to no guidance on effective leadership and teamwork principles necessary to be an effective member of a trauma team within these courses [3]. Utilization of concepts espoused in Crisis Resource Management (CRM) may aid in the acquisition of basic skills required to be an effective member of a trauma team [4]. This is especially useful in an environment like a trauma bay where there are a variety of inter-professional teams who may have never worked together and are therefore unaware of each other skills and abilities. Medical simulation has demonstrated effectiveness as a teaching methodology to train in CRM principles and error management training, which is often missing in residency curriculum for interns throughout the United States [5-6].

The concepts of vicarious error management training (EMT) and error-based learning provide an additional avenue in the effective training of novice learners [7]. EMT allows interns to gain insight through open discussion of common errors, focusing on how to avoid them and the subsequent management of their consequences [7]. An emphasis on discussing common errors allows interns to become more adept at correcting them and ultimately provides the emotional stability to adjust their management when errors are made in the clinical setting [8]. These skills can be reinforced using deliberate practice (DP) methods. Deliberate practice supports the idea that educational techniques should be repetitive with close scrutiny by content experts who provide immediate detailed feedback on enhancing performance [9]. Through DP, the learner seeks to improve their performance and knowledge by preventing automatism and raising their own standards [10].

In this study, we describe the creation of an interdisciplinary boot camp in which interns' basic knowledge, level of confidence, and teamwork skills are assessed. Our interdisciplinary definition was based on the Oregon Health & Science University Interprofessional Initiative Glossary. Interdisciplinary training is defined as different disciplines working collaboratively toward a common purpose [11]. The primary goal of the study was to create a curriculum focused on communication and teamwork skills as a foundational skill set necessary for effective management of acute trauma patients.

## Materials and methods

### Study and boot camp curriculum design

**Figure 1:**
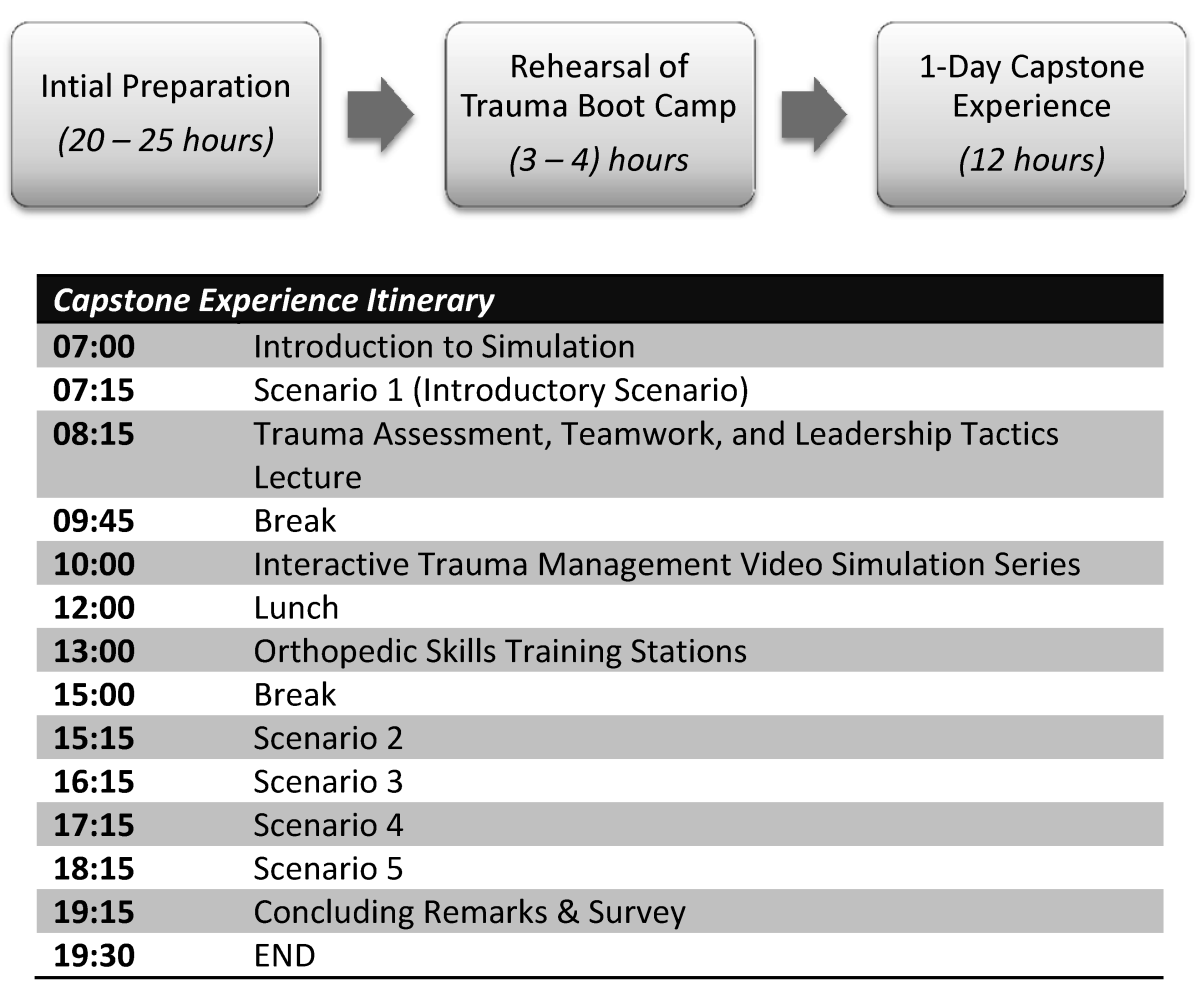
Schedule outlining the preparation and execution of the trauma boot camp

A one-day boot camp curriculum was designed for interns of various specialties who are part of the trauma response team at our institution. Our institution trauma response team consists of attending physicians, resident physicians, and nursing and respiratory therapists. However, education in the simulation laboratory was targeted at interns. The main goal of the boot camp was to assess and teach team dynamics during high-fidelity simulated trauma cases. The curriculum consisted of a one-day, twelve-hour experience, which included trauma patient simulation stations; content expert lectures from trauma surgeons, orthopedic surgeons, and emergency medicine physicians; group discussion of video demonstrations; and skill development workstations (Figure 1). Prior to the execution of the trauma boot camp, 20-25 hours of initial preparation from the Simulation and Trauma Surgery faculty was required for development of the curriculum, surveys, questionnaires, and simulation cases. After initial preparation, all participating staff performed a rehearsal of the trauma boot camp to assure simulation cases were executed without difficulties and all necessary equipment was available (3-4 hours). Overall, preparation and execution of the curriculum took around 30-35 hours.

### One-cay capstone experience

**Figure 2:**
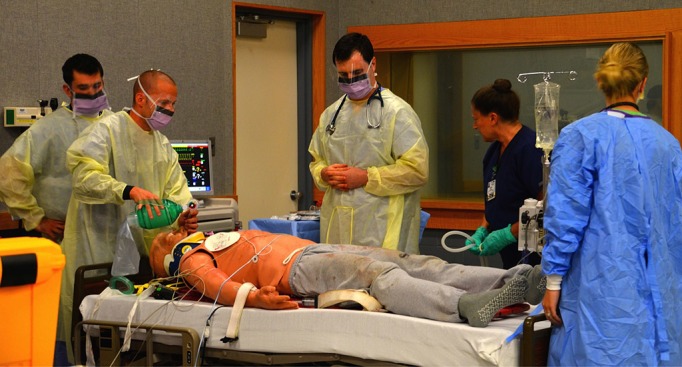
Trauma boot camp Interns managing a simulated trauma patient during a one day trauma boot camp

A total of 15 interns from different specialties (Surgery-4, Orthopedics-7, Urology-2, Emergency Medicine-2) participated in the interdisciplinary trauma boot camp. None of the interns had completed ATLS prior to participating in this curriculum. Our twelve-hour boot camp consisted of five parts (Figure 1). At the onset of the boot camp, interns were provided an introduction to the simulation environment to orient themselves with the simulation mannequin, resuscitation bay, available equipment, and ancillary staff. Each resuscitation bay was stocked in a similar fashion to the hospital’s resuscitation bays (Figure 2). Interns were informed that the simulation lab was a learning environment and were encouraged to manage the simulated scenarios as realistically as possible. They then participated in an introductory dual-trauma simulated scenario with both a standardized patient and a high-fidelity mannequin patient. Residents were initially presented with a single patient involved in a motor vehicle collision. The patient presented with a low Glasgow Coma Scale (GCS) score secondary to an intracranial hemorrhage. Three minutes into the simulation, a second simulated trauma victim (standardized patient actor) presented via EMS with a traumatic lower extremity amputation. The interdisciplinary team had to subsequently divide into two teams and manage both critical patients with limited resources and personnel. This provided the faculty an opportunity to assess residents' baseline CRM aptitude and leadership skills. Subsequently, they received an extensive debriefing with a major emphasis on teamwork, leadership skills, and CRM principles by our simulation medical director.

**Figure 3:**
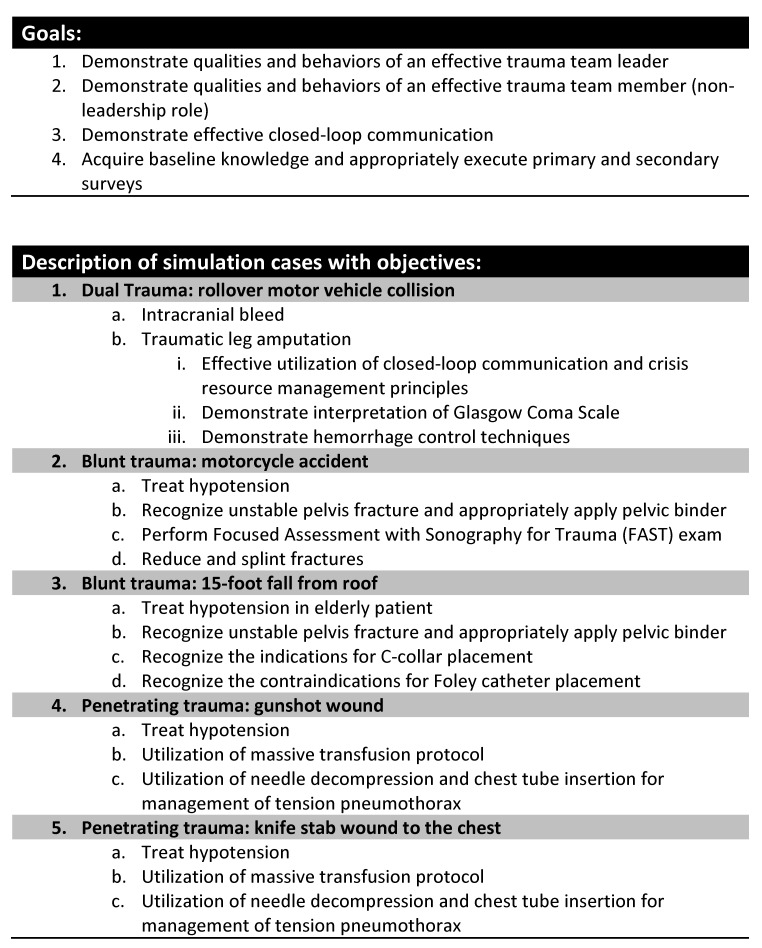
Objectives and outline of simulation cases

Interns then received an introductory content expert lecture on the assessment and management of primary and secondary survey by our trauma medical director. This lecture did not include advanced assessments or treatments. Interns watched a series of recorded video trauma simulations with varying degrees of intentional errors to facilitate interactive discussion of errors and mismanagement in the trauma bay (vicarious error management). This video series was created and the discussion conducted by one of our trauma surgeons and our simulation medical director. In the afternoon session, participants took part in three orthopedic skill stations taught by Orthopedic Surgeons. The boot camp culminated in a series of simulated trauma cases (4, one-hour sessions including debriefing). The two blunt cases and the two penetrating cases (4 different stems) had identical pathophysiology injury patterns and required essentially identical management (Figure 3). Interns were divided into two groups of 7-8 participants. Structured debriefing was provided after each scenario by faculty present at the boot camp. All simulated cases, including the introduction, were recorded for review and evaluation using a validated evaluation tool of teamwork and leadership skills for trauma (Non-Technical Skills, NOTECHs) [12]. Three trained ATLS physicians who were not involved in the study evaluated the simulation cases for leadership and teamwork skills.

### Survey questionnaire

Pre- and post-intervention confidence surveys were administered to each of the participants of the trauma boot camp prior to beginning the curriculum as well as at the conclusion of the training. The pre- and post-intervention confidence survey consisted of 15 questions. A 15-question, pre- and post-cognitive questionnaire was internally developed by trauma faculty to assess interns’ baseline and acquired knowledge. This was a quality assurance project and was reviewed by the IRB. It was considered exempt, as it did not meet the criteria for human subject research.

## Results

**Figure 4:**
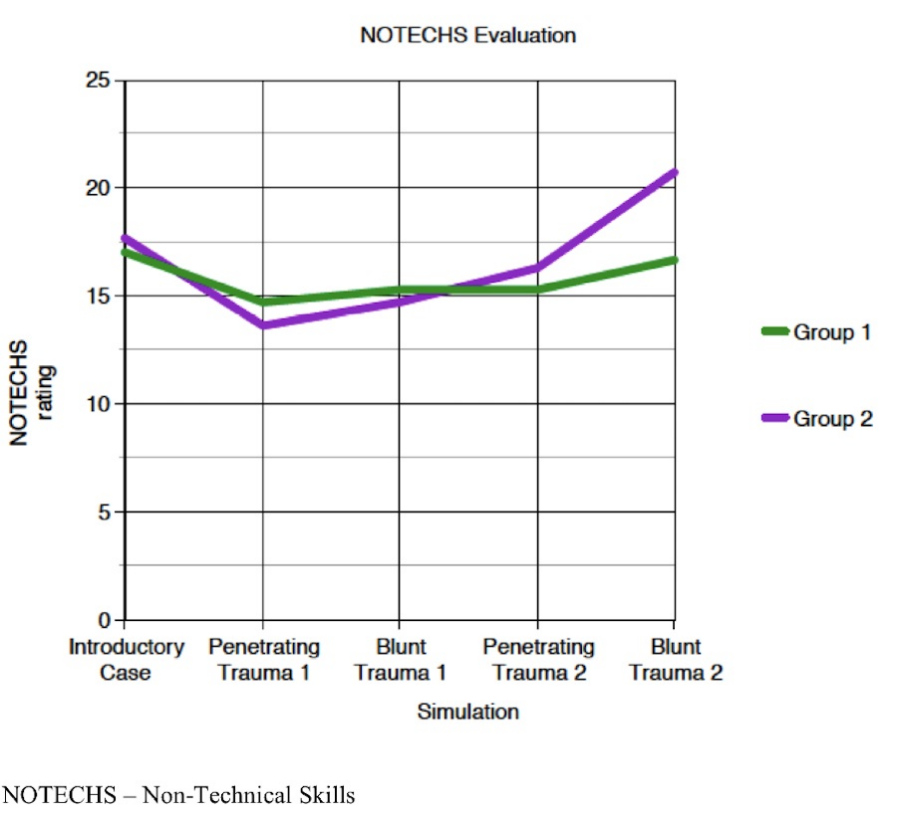
Non-Technical Skills (NOTECHS) evaluations

The results of this study showed that upon entering this boot camp, residents had an overall confidence score of 3.2 (SD = 0.44) (1-5 scale, 5 being the greatest) in the management of trauma cases. Following the boot camp, confidence scores significantly increased to 3.9 (SD= 0.34) (p<0.0001) (Table 1). They received a mean score of 9.93 (66%) on the pre-cognitive exam (0-15), which subsequently increased to 11.13 (74%) on the post exam. NOTECHS evaluations had an average score of 17 and 17.7 (5-25) for groups one and two, respectively, during their baseline trauma simulation. Group 1’s final simulation performance decreased to 16.7. Group 2’s final simulation performance increased to 20.7 (Figure 4).

**Table 1:** Confidence Score Results SD – standard deviation

	Pre-test Avg (SD)	Post-test Avg (SD)	P-value
1. I understand how a trauma activation should be run	3.2 (0.56)	4.27 (0.46)	<0.0001*
2. I understand my role and other team members roles’ during a trauma activation	3.27 (0.80)	4.21 (0.43)	0.0004*
3. I feel confident I can recognize when a trauma patient should be intubated in the trauma bay	2.93 (0.59)	3.87 (0.52)	<0.0001*
4. I am confident I could be the airway person during a trauma activation	2.6 (0.74)	3.27 (0.80)	0.0243*
5. I am confident determining the Glasgow coma scale and can use it to guide the care of a patient	2.93 (0.80)	3.13 (0.74)	0.4831
6. I understand the use of a backboard and cervical collar for a trauma patient	3.53 (0.52)	4.07 (0.26)	0.003*
7. I understand when it is appropriate to transfuse blood products during a trauma activation	3.27 (0.59)	4.07 (0.26)	0.0001*
8. I understand when a trauma patient is hemodynamically unstable	3.6 (0.51)	4.07 (0.46)	0.0131*
9. I am confident I could lead a trauma activation utilizing key principles of crisis resource management	2.47 (0.74)	3.6 (0.63)	0.0001*
10. I am confident I can demonstrate effective closed loop communication during a trauma activation	3.8 (0.68)	3.93 (0.46)	0.5446
11. I am confident performing the primary and secondary surveys of trauma	3.13 (0.99)	4.07 (0.26)	0.0014*
12. I am confident I could be the primary resident during a trauma activation, performing most of the physical exam	2.8 (0.94)	3.93 (0.59)	0.0005*
13. I am confident I could be the secondary resident during a trauma activation, assisting the primary resident by removing clothing and helping to logroll the patient	4.13 (0.99)	4.27 (0.59)	0.6417
14. I am confident I can adequately reduce/splint fractures in a trauma setting	3.07 (1.44)	3.67 (1.23)	0.23
15. I am confident I know the indications and contraindications of placing a Foley catheter	3.27 (0.70)	4.07 (0.80)	0.0069*

## Discussion

Despite the many strengths of the ATLS program, the goal of the study was to create a supplementary curriculum that addresses the main shortcoming within the course: the deficiency of teamwork and leadership training. The boot camp curriculum was designed to increase interns’ CRM skills, leadership principles, and closed loop communication in various trauma scenarios. Despite having ATLS training, residents often struggle within leadership roles, especially with an unfamiliar team [13]. The boot camp included participants from various specialties who had to efficiently delegate tasks, assign team roles, and assess the situation in order to achieve a positive patient outcome. Each of the five scenarios not only tested interns’ baseline trauma knowledge, but also examined their ability to utilize CRM principles. Rotating leadership positions provided opportunities for participants to fulfill one of the multiple roles present on a trauma team. This allowed interns to participate in both leadership and non-leadership positions and cultivate skills that create a high-functioning trauma team.

At the culmination of the study, various trends were observed. There was a significant increase in the overall confidence level of interns in role delegation, leadership, CRM principles, and in the performance of primary and secondary surveys. These trends can be attributed to reinforcement of CRM concepts under the guidance of faculty during repeated debriefings. EMT videos provided a fruitful discussion on appropriate and inappropriate leadership, role delegation, communication, and team dynamics. We believe the rise in confidence may also be attributed to the interns’ self-recognition of strengths and weaknesses as a result of participation in high intensity simulated trauma scenarios. The participants were unaware that both blunt cases and penetrating cases had identical pathophysiology branch points. This provided a deliberate practice environment that allowed true, innate decision making, preventing automaticity that can occur as a result of informing the interns they were managing the same case. This allowed faculty an opportunity to assess the effectiveness of the simulation and subsequent debriefing.

Interestingly, there were fields in which significant increases were not seen. Interns started and ended the boot camp with about the same level of confidence when it came to determining and effectively using the GCS. Since the use of GCS was introduced only in the trauma lecture, the small change in confidence could be attributed to the limited time given to discuss and apply this concept. Confidence levels in orthopedic splinting/reduction skills also did not improve significantly, despite the two hours of training with senior orthopedic residents, suggesting that more extensive training is still needed in this particular skill. The effective use of closed-loop communication displayed an interesting trend. Initially, interns had high confidence levels, but by the culmination of the boot camp, displayed only a negligible increase in levels. Evaluation of performance by content experts demonstrated the interns had lower scores than initially self-reported with regard to teamwork and communication skills. This could be attributed to an overconfidence or lack of understanding of the concepts of closed-loop communication [14]. By the final simulation, the NOTECHS results did not match the interns’ level of confidence in teamwork and communication skills. This suggests that CRM concepts need continuous practice and development to achieve proficiency. This discrepancy could also be explained by participants' individual personality traits, as the leadership position was interchanged in each simulation scenario allowing each participant to be the leader in only one scenario (if at all). 

The NOTECHS data obtained demonstrated an overall increase in one team’s performance and no statistical significant change in the other (Figure 4). Despite the combination of didactic lecture, vicarious EMT video discussion, deliberate practice training, and post-simulation debriefing, there was no significant improvement in performance after this intensive curriculum. Despite our extensive training, we are not surprised by the expert panel evaluation of the interns’ performance. We believe ultimately that effective performance of CRM principles in acute trauma settings requires additional simulation training and deliberate practice methodologies.

There are previously published studies with similar curricula. Miyasaka et al. developed a three-day program focused on trauma and surgical, critical-care patients for surgery residents [15]. Their training included hands-on skills, didactics and simulation scenarios. Overall, surgery residents showed an increased confidence after the three-day training. Baker et al. developed a curriculum that targeted undergraduate students [16]. CRM concepts were taught with TeamSTEPPS® training. Concepts were subsequently practiced in trauma scenarios. The training improved participants’ team leadership, situational monitoring, and overall communication. Our curriculum included all interns who participate in a trauma service activation in a multidisciplinary fashion. It was designed to be a one-day boot camp with the idea of providing communication and leadership training to interns before starting their trauma rotation. This study provides a specific outline for educators with defined goals and objectives that can be used as a supplement to the existing ATLS course. 

### Limitations

This study has several limitations. First, we utilized a non-validated confidence survey and multiple-choice test to collect data. Although our sample consisted of residents from two institutions, the sample size itself was small. Lastly, the specialties observed during the boot camp reflected our own institution’s staffing model for trauma activations.

## Conclusions

At the end of the intensive, one-day trauma boot camp, interns demonstrated significant improvement in self-reported confidence of CRM concepts, role delegation, leadership, and performance of primary and secondary surveys. Despite the intensive twelve-hour curriculum, there was no significant improvement in overall teamwork and leadership performance during simulated cases as rated by content experts. Our boot camp curriculum offers educators a unique framework to which they can apply to their own training program as a foundation for effective leadership and CRM principles for novice residents.
